# Breaking bad news education for emergency medicine residents: A novel training module using simulation with the SPIKES protocol

**DOI:** 10.4103/0974-2700.70760

**Published:** 2010

**Authors:** Inchoel Park, Amit Gupta, Kaivon Mandani, Laura Haubner, Brad Peckler

**Affiliations:** Department of Emergency Medicine, USA; 1 Department of Emergency Medicine, Pediatrics, University of South Florida, Tampa General Hospital, Tampa, FL, USA

**Keywords:** Delivering bad news, education, simulation

## Abstract

Breaking bad news (BBN) in the emergency department (ED) is a common occurrence. This is especially true for an emergency physician (EP) as there is little time to prepare for the event and likely little or no knowledge of the patients or family background information. At our institution, there is no formal training for EP residents in delivering bad news. We felt teaching emergency medicine residents these communication skills should be an important part of their educational curriculum. We describe our experience with a defined educational program designed to educate and improve physician’s confidence and competence in bad news and death notification. A regularly scheduled 5-h grand rounds conference time frame was dedicated to the education of EM residents about BBN. A multidisciplinary approach was taken to broaden the prospective of the participants. The course included lectures from different specialties, role playing for three short scenarios in different capacities, and hi-fidelity simulation cases with volatile psychosocial issues and stressors. Participants were asked to fill out a self-efficacy form and evaluation sheets. Fourteen emergency residents participated and all thought that this education is necessary. The mean score of usefulness is 4.73 on a Likert Scale from 1 to 5. The simulation part was thought to be the most useful (43%), with role play 14%, and lecture 7%. We believe that teaching physicians to BBN in a controlled environment is a good use of educational time and an important procedure that EP must learn.

## INTRODUCTION

Breaking bad news (BBN) in the emergency department (ED) is a common occurrence. Sudden or unexpected diagnoses or deaths happen frequently in the ED. In 2005, there were 287,000 ED deaths in USA.[1] BBN with empathy and compassion is an important and difficult task for any physician. In the best of circumstances, a physician should be able to communicate effectively, should know all about their patients’ problems, and take a patient-/family-centered approach.[2] The ability to demonstrate empathy and caring can be as important as diagnostic and treatment skills.[3] This is especially vital for an emergency physician (EP) as there is little time to prepare for the event, and there is likely little or no knowledge of the patients’ or any family background information.

At our institution, there is no formal training for ED residents in BBN. We felt teaching emergency medicine residents these communication skills should be an important part of their educational curriculum. Despite the importance of this interaction, there are few reported formal training courses that are focused on EPs, nor are there universal programs to teach residents/fellows on how to convey this information effectively. In medical schools, such courses are mostly taught through didactic lectures or seminars.[4] Postgraduate courses are usually consist of 1 or 2 days of teaching and include lectures, videos, and role play.[4,5] These formats are lengthy and not practical for emergency residents. One such course is the “SPIKES” model.[6] The acronym SPIKES (Set the stage, Perception, Inform, Knowledge, Empathy, and Summarize) can be adapted to be a useful tool for an EP when BBN. This is a six-step process [Table 1] that focuses on gathering information, transmitting the medical information, providing support, and summarizing the information. The workshop described in this manuscript utilized a regularly scheduled grand rounds lecture time focusing on BBN using the “SPIKES” model along with simulation. This study describes an educational intervention designed to educate and improve physician’s confidence and competence in delivery of bad news and death notification.

**Table 1:** S-P-I-K-E-S competence Form – SHORT FORM

Directions: Please indicate whether the physician completed the stated actions, with Y = completed (Yes) or N = did not complete (No)
S – Set the stage
1. Clearly introduced herself/himself
2. Clearly stated his/her role in the care of the patient
P – Perception
3. Determined the level of knowledge the survivors possessed prior to their arrival in the waiting room
4. Took note of the news receiver’s vocabulary
I – Inform
5. Briefly indicated the chronology of events leading up to the death of the patient
6. Used language appropriate for the survivor’s culture and educational level
7. Avoided using euphemisms
K – Knowledge
8. Allowed the survivor to react to the information and ask questions or express concerns
9. Answered ALL questions in an appropriate manner
E – Empathy
10. Used proper statements to show concern for the grieving
11. Validated emotions of the grieving
S – Summary and Strategy
12. Avoided showing any physician guilt for the loss/poor prognosis
13. Established personal availability to answer questions for the survivor at a later date
14. Ended the discussion and departed in an appropriate manner

## WORKSHOP DESCRIPTION

A regularly scheduled 5-h grand rounds conference time frame was dedicated to the education of EM residents about BBN. Five first-year, 4 second-year, and 5 third-year emergency medicine residents participated in the workshop.

The workshop was adapted after a training course module using the GRIEV_ING^©^ protocol developed for EPs and residents under a grant from the US Department of Justice.[7] A multidisciplinary educational team approach was taken to broaden the perspective of the participants. Our course consisted of an overview lecture on the challenges of delivering bad news in the ED by an EP. This lecture focused on specifics in the ED and gave a detailed instruction on the “SPIKES” protocol. A 20-min video[8] describing patients’ and senior physicians’ feelings on the subject, was then shown to the participants. The video was an emotional and clinical introduction to this topic. There is a Chaplain residency at our institution, which is an integral part of the trauma resuscitation protocol. The director of the residency discussed the role of effective communication skills and reflective listening.[9] He also stressed the Chaplain’s role in delivering BBN and how to use them effectively. The aspect of BBN involving children was presented by a neonatologist. The final lecture was delivered by a surgical oncologist who herself was a cancer survivor. The focus of her lecture was on how she delivers bad news and her feelings after receiving a poor prognosis herself. All the lectures stressed empathetic communication during death and bad news notification and the necessary information to give to the family.

The next session consisted of role play using three short scenarios. The first case involved a man who was assaulted while visiting for his college reunion and dies from his injuries. The survivor was his wife who was with him at the time of the incident. The second case involves a single female police officer, who was the mother of three children, was killed while in pursuit of a drunken driver. The survivors were her friend and a captain. The last case involved the death of a three-year-old boy who died in a house fire, and the survivor was the litigious distraught father. The participants played the physician, survivor, or observer of the interaction in a case, and then rotated in the next scenario. The “physician” goal was to practice the skill of empathetic communication and use the SPIKES protocol to deliver the death or bad news notification to the survivor. The “survivor,” i.e. patient/relative reacted to the news provided by the physician. The “observer” used the SPIKES Competence Short Form as a checklist as they observed the physician’s skills [Table 1]. The observers took notes and provided constructive feedback to both physician and survivor with a short debrief. Attending EPs, patient representatives, and Chaplains were also present in the small groups and added to the debrief discussion.

The final session of the workshop was done in the simulation lab with live video broadcast to the workshop audience. The cases were designed to create a high-pressure environment and have volatile psychosocial issues and stressors. The scenarios included a standardized patient as the recipient of the bad news, an actual ED nurse, social worker, Chaplain, and a patient representative playing their actual clinical roles. The first case involved a car accident with the driver being an intoxicated teenager who is injured but will need to go to the operating room and his passenger, an exchange student from Germany who was staying with the driver’s family (a widowed parent); the student is killed in the accident. The second case was a non-helmeted 12-year-old child on a bicycle that was struck by a car and sustains severe polytrauma and eventually dies in the ED. The guardian is the grandmother who is watching the child while the parents are on a vacation. The third case involved an elderly woman who lived alone who was the victim of a break-in and assault in her house. Her daughter who has not seen her in a few weeks is the one who discovers her at home and is present in the hospital. The patient dies in the ED from her injuries. An extensive debrief was performed afterward with video recall.

A course survey evaluated the activity as a whole and the quality and usefulness of each session. In the self-efficacy form, there were items similar to the SPIKES forms. After the workshop, all participants were asked to fill out the evaluation sheets.

## EVALUATION

**Figure 1:**
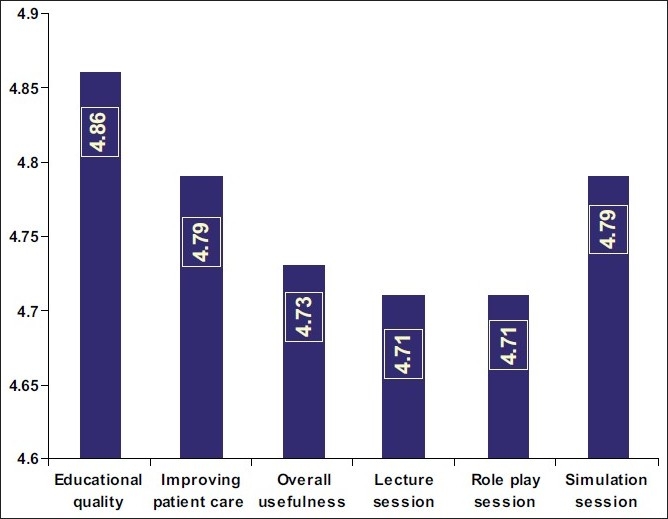
Comparative chart of the mean Likert scores. Likert scale (1, lowest to 5, highest) given to each aspect of the workshop

There were 14 emergency residents who participated. All participants thought that the educational experience was necessary. The result of the usefulness of this workshop was evaluated using a 5-point Likert Scale (1, worst to 5, best). Figure 1 represents the mean scores given to the educational quality and overall usefulness of the various sessions. The participants thought that patient care would improve after this educational experience with a mean Likert score of 4.79. The overall usefulness of the course was scored 4.73.

**Figure 2:**
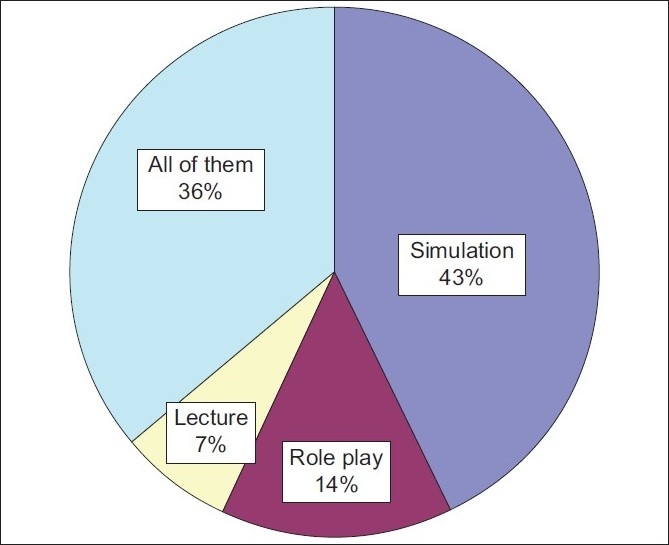
The most useful sessions

Figure 2 shows the distribution of the most useful session as rated by the participants. Among the participants, 43% thought that the session on simulation was the most useful, as compared to role play 14% and lectures 7%. Of the participants, 36% found all the sessions equally useful. All the participants agreed that the course should be continued as part of their annual regular academic activity.

## DISCUSSION

Teaching behaviors to physicians is very different and more difficult than teaching knowledge. The skill of delivering bad news to patient or his/her family is an important task that an EP must perform. We believe that this is a skill that should be approached like any important high impact medical or surgical procedure.[10,11] Other specialties including oncology, neurosciences, psychology, and nursing area have formal program to teach how to deliver bad news.[12–16]

The SPIKES protocol was developed in 2000 by an oncologist[6] to train providers in delivering bad news. In 2005, the GRIEV_ING educational intervention was developed and tried by emergency physicians.[15,17] The GRIEV_ING interventions consisted of a 2-h educational session composed of a didactic experience with small group role play. The results of this study revealed a significant increase in confidence, and competence scores in residents’ skill of death notification from pre- to postintervention assessments.

In our workshop, we used didactic lectures, small group role play, and simulation. The SPIKES protocol was used to deliver bad news. The small group role play from the three different perspectives was useful to the participants. The objective was to expose them to the giving and receiving of bad news and to observe the interactions. We felt this would be a useful way to help process and practice the information in a short period. Simulation education can be more realistic than the other education tools.[18,19] A high fidelity simulator and standardized patient can create for the learner “suspension of disbelief.” The debriefing sessions after the simulation cases were intense and extremely emotional at times. It was not surprising that participants found this session the most helpful as the multidisciplinary approach during the debriefing sessions generated many interesting discussion topics for the group.

In a real BBN situation, it would be ethically challenging to let a novice with minimal training, to deliver the information just as it would be to let a person perform a medical procedure without any formal training. It is also worth noting that if informed improperly, families may suffer from long-term emotional consequences and pathologic grief reactions.[20] All participants felt that this educational intervention was helpful and recommended that it should be repeated each year as a part of formal EM curriculum.

## CONCLUSION

It is unavoidable that in the ED, there will be many opportunities to deliver bad news to the patient or the family. It is also likely and different from other specialties that an EP has no significant past relationship with the patient or family. Lastly, EM residents untrained and unskilled in BBN will likely then be left to learn this skill through practice on actual patients’ families. Family members in ED are in a stressful situation, limited psychological preparation, do not know the physician, and they have little or no contact with either before or afterward. Often the EPs will be called to do this vitally important duty immediately after an emotionally exhausting failed resuscitation. It is our suspicion that if delivering bad news is done improperly, it can create ill will among the family and be detrimental to the grieving process. We believe this would be bad medicine.

We believe that the workshop was a worthwhile use of valuable educational time. Using simulation as an educational tool along with the SPIKES protocol and small group role play is a novel and efficient module to teach EPs how to break bad news in the stressful environments of an emergency room.
